# Supplementary material for: Enhanced Lung Cancer Detection Using a Combined Ratio of Antigen–Autoantibody Immune Complexes against CYFRA 21-1 and p53
Source: Cancers (Basel). 2024 Jul 26;16(15):2661. doi: 10.3390/cancers16152661 (PMC11312164; doi:10.3390/cancers16152661)
Supplement: Supplementary file 1 [file cancers-16-02661-s001.zip › cancers-3108113-supplementary.pdf]

**Table S1**. The TNM stage of non-small cell lung cancer patients ($n$=90).

| **Characteristics** | **Lung cancer (n = 90)** |
|---|---|
| Stage | |
| Non-small cell lung cancer | 90 |
| -TNM Stage I | 39 |
| IA | 26 |
| IB | 13 |
| -TNM stage II | 12 |
| IIA | 3 |
| IIB | 9 |
| -TNM stage III | 20 |
| IIIA | 12 |
| IIIB | 5 |
| IIIC | 3 |
| -TNM stage IV | 19 |
| IVA | 5 |
| IVB | 4 |

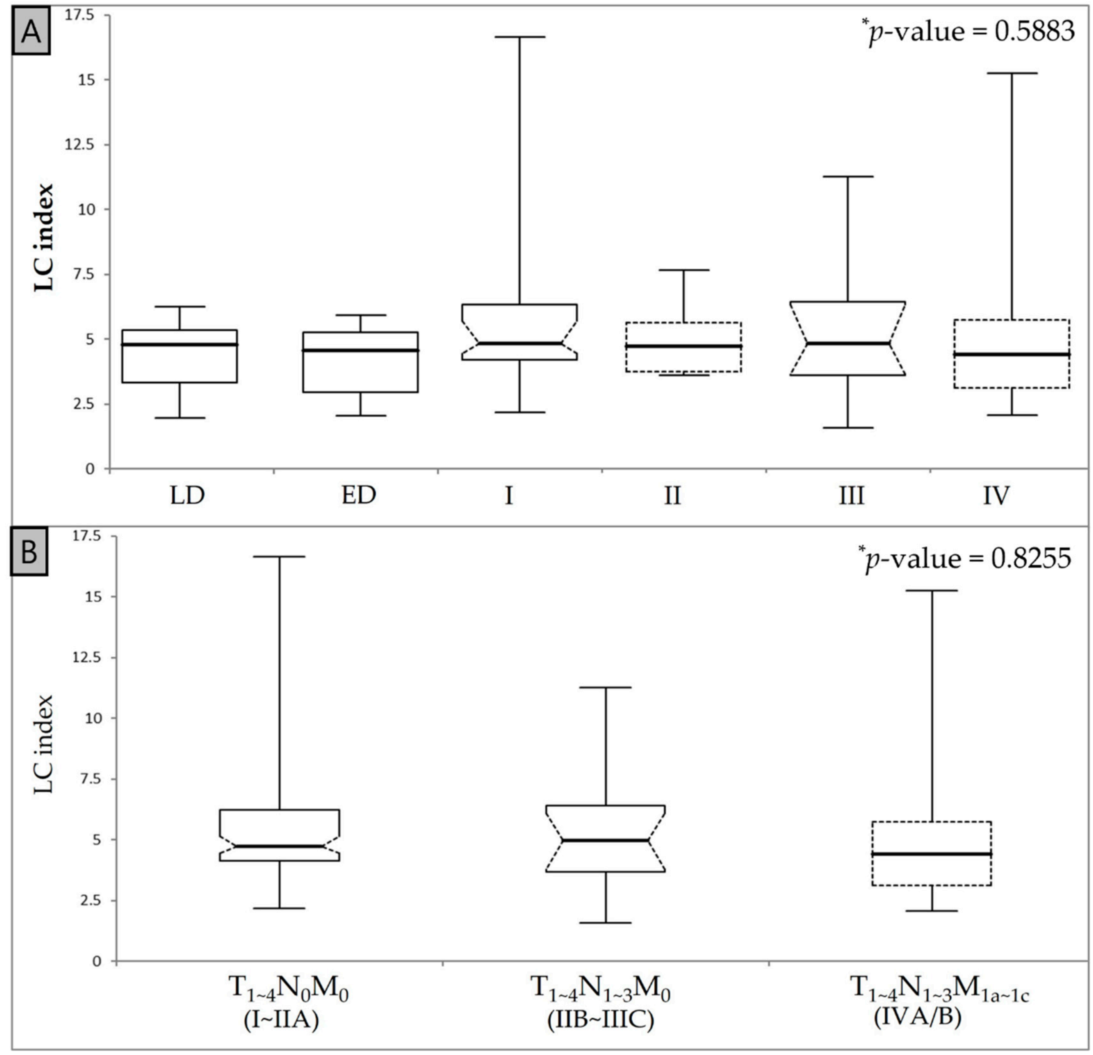


**Figure S1.** Levels of the LC index according to lung cancer stages. (A) Levels of the LC index of the NSCLC and SCLC stages (*n* = 100); (B) Levels of the LC index according to TNM stages, where three groups are divided by their T/N/M status of NSCLC patients (*n* = 90). **p*-value was estimated by the Kruskal–Wallis test. No significant difference was observed between each stage or stage group (all, *p*-value = 1.0000). Abbreviations: ED, extensive disease; LD, limited disease
